# Assessing hypothalamic pituitary gonadal function in reproductive disorders

**DOI:** 10.1042/CS20220146

**Published:** 2023-06-05

**Authors:** Kanyada Koysombat, Waljit S. Dhillo, Ali Abbara

**Affiliations:** 1 Section of Investigative Medicine, Imperial College London, London, United Kingdom; 2 Department of Endocrinology, Imperial College Healthcare NHS Trust, London, United Kingdom

**Keywords:** Congenital hypogonadotrophic hypogonadism, Constitutional delay of growth and puberty, delayed puberty, Functional hypothalamic amenorrhoea, hypothalamo-pituitary-gonadal axis, Polycystic ovary syndrome

## Abstract

Reproductive conditions secondary to disorders of the hypothalamic–pituitary–gonadal (HPG) axis are common and are associated with important health implications and considerable psychosocial impact. Basal and dynamic tests enable interrogation of individual components of the HPG axis, facilitating diagnosis and understanding of the pathophysiology of reproductive disorders. Onset of puberty is controlled by hypothalamic gonadotrophin-releasing hormone (GnRH) neuronal function. To date, a dynamic test of hypothalamic function is not yet available. Therefore, accurate differentiation of pubertal disorders such as constitutional delay of growth and puberty (CDGP) and congenital hypogonadotrophic hypogonadism (CHH) as causes of delayed puberty is challenging due to similar clinical presentations and hormonal profiles. Likewise, although the two commonest reproductive disorders in women, polycystic ovary syndrome (PCOS) and functional hypothalamic amenorrhoea (FHA) have disparate hypothalamic function, oligo/amenorrhoea frequently poses a diagnostic conundrum owing to the overlap in the criteria used to define both conditions. This review aims to describe pubertal and reproductive disorders secondary to pathologies affecting the HPG axis. Challenges encountered in clinical practice in differentiating pubertal and reproductive conditions are reviewed in conjunction with the utility of baseline and dynamic endocrine tests to interrogate specific components of the HPG axis. We also highlight putative hypothalamic, pituitary, and gonadal markers in development that could improve the diagnosis of patients presenting with disorders of puberty or reproduction.

## Introduction

The hypothalamic–pituitary–gonadal (HPG) axis describes the regulation of the reproductive hormones by the hypothalamus, anterior pituitary gland, and gonads. At the level of the hypothalamus, gonadotrophin-releasing hormone (GnRH) is secreted in a pulsatile manner into the hypophyseal-portal circulation. GnRH acts via the GnRH receptor (GnRHR) on pituitary gonadotrophs to stimulate the synthesis and secretion of luteinising hormone (LH) and follicle-stimulating hormone (FSH), which subsequently stimulate gonadal steroidogenesis and regulate gametogenesis.

In females, feedback from oestradiol (E2) on the HPG axis is critical for the regulation of major events in the menstrual cycle, including follicular growth, the mid-cycle LH surge and resultant ovulation, and formation of the corpus luteum. The absence of key receptors, such as oestrogen receptor α (ERα) [1], progesterone, and leptin receptors [2] on GnRH neurons suggested the presence of afferent, intermediary neurons that provide integrated neuroendocrine, sex-steroid and metabolic feedback on GnRH neurons. Kisspeptin neurons have direct contacts with GnRH neuronal perikarya and dendrons [3] and have emerged as key regulators of GnRH neuronal activity and subsequent pulsatile GnRH secretion [4].

Kisspeptin is a family of hypothalamic neuropeptides encoded by the *KISS1* gene in humans [5]. Seminal papers have demonstrated that inactivating variants of *KISS1* and its receptor, encoded by *KISS1R*, result in pubertal delay and congenital hypogonadotrophic hypogonadism (CHH) [6–8] characterised by failure of GnRH secretion. Conversely, activating variants in *KISS1R* and *KISS1* caused central precocious puberty (CPP) [8,9]. These observations first indicated the crucial role of kisspeptin in the timing of puberty, reproduction, and regulation of the HPG axis.

Kisspeptin neurons are expressed in two discrete hypothalamic nuclei, namely the arcuate nucleus (ARC) (homologous to the infundibular nucleus in humans) and the rostral periventricular area of the third ventricle (RP3V) [10]. In several animal species, kisspeptin neurons in the ARC co-express neurokinin B and dynorphin providing autocrine/paracrine regulation of GnRH secretion and are thus collectively known as ‘KNDy neurons’ [11]. During the follicular phase, low E2 levels exerts negative feedback on ARC kisspeptin neurons resulting in the maintenance of pulsatile GnRH (and in turn LH) secretion. Conversely, during the preovulatory stage, high E2 induces positive feedback on anteroventral periventricular nucleus (AVPV) kisspeptin neurons in the RP3V to instigate the midcycle GnRH/LH surge and resultant ovulation [10].

GnRH primarily acts on the pituitary gonadotrophs to drive synthesis and secretion of LH and FSH. Pulsatile GnRH secretion is critical to the stimulation of pituitary gonadotrophs; indeed, sustained GnRH administration results in down-regulation of the GnRH receptor and decreased gonadotrophin secretion [12]. Furthermore, GnRH pulse amplitude and frequency are critical determinants of gonadotrophin synthesis and secretion; fast pulse-frequencies (>1 pulse per hr) favour LH secretion through increased *LH-α* and *LH-β* transcription, whereas slow pulse-frequencies (<1 pulse per 2–3 h) favour FSH secretion through increased *FSH-β* transcription [13]. LH and FSH are glycoprotein hormones that stimulate spermatogenesis, folliculogenesis, ovulation, and production of gonadal sex-steroids. In humans, peripheral sampling for GnRH cannot be conducted to reflect central hypothalamic GnRH neuronal activity. Although both LH and FSH secretion are controlled by pulsatile GnRH secretion, FSH is less susceptible to marked fluctuations with each GnRH pulse compared with LH owing to its longer half-life (1 h vs 20 min). Therefore, measurement of LH pulsatility is used instead to reflect GnRH pulsatility.

This review first describes conditions affecting puberty and reproductive health secondary to pathologies affecting the HPG axis. We then summarise challenges encountered in clinical practice in differentiating pubertal and reproductive conditions that centre around the HPG axis and review baseline and dynamic endocrine tests that can be used to interrogate hypothalamic, pituitary, and gonadal function.

## Disorders of puberty

During foetal life and infancy, there is a period of transient HPG axis activation (termed ‘mini-puberty’), followed by relative quiescence of the HPG axis until the onset of puberty [14]. The mechanism for maintenance of this quiescence has been a long-standing mystery, although the discovery of the makorin RING finger protein 3 (MKRN3) and its role in inhibition of *KISS1* and *TAC3* transcription has begun to shed light on this [15]. The reawakening of pulsatile GnRH secretion and activation of the downstream reproductive endocrine axis induces the onset of puberty, which is characterised by the acquisition of secondary sexual characteristics and reproductive capacity [14].

### Delayed puberty

Delayed puberty is defined as the absence of breast development in girls and testicular enlargement in boys beyond 4 ml at an age that is >2 standard deviations (SD) later than the population mean [16]. Although the precise ages depend on the population studied, pubertal onset occurs in most (95%) adolescents between the ages of 8.5–13 years in girls and 9–13.5 years in boys [17]. Delayed puberty is more common in boys and usually occurs due to constitutional delay of growth and puberty (CDGP). CDGP is the commonest cause of pubertal delay affecting 60–80% of boys and 30–55% of girls with delayed puberty [18], whereby puberty will commence spontaneously with conservative management [16].

An important alternate cause of delayed puberty is CHH found in 10–20% of cases of delayed puberty, characterised by failure of GnRH secretion or action resulting in absent or incomplete puberty [18]. To date, more than 50 genes have been shown to contribute to the pathogenesis of CHH [19], the identification of which have furthered our understanding of key pathways in the HPG axis. CHH can result from variants in genes that regulate GnRH neuronal development, migration and maturation such as *ANOS1*, *PROK2, PROK2R, FGFR1* and *CHD7* and those involved in GnRH function including *KISS1, KISS1R, TAC3, TAC3R, GNRH1*/*GNRHR*, *FSHB* and *LHB* [19]. Administration of exogenous GnRH to patients with loss-of-function *KISS1R* variants and *Kiss1r* knockout mice models (which recapitulated the phenotypes observed in human), rescues gonadotrophin secretion [7]. Loss-of-function variants in the *TAC3* and *TAC3R* genes, which encode neurokinin-B and its receptor, respectively, can also cause CHH, highlighting the importance of KNDy neurons in the regulation of GnRH secretion and puberty.

Although CHH can be associated with red flag symptoms such as anosmia/hyposmia, cryptorchidism, micropenis, bimanual synkinesia, hearing loss, and cleft palate, these signs are not always present [18]. This, together with overlapping biochemical profile (hypogonadotrophic hypogonadism), makes the distinction between CDGP and CHH challenging, frequently resulting in diagnostic delay [20]. Timely and accurate diagnosis is crucial, as although CDGP can be managed conservatively with reassurance, patients with CHH could benefit from timely pubertal induction to safeguard future reproductive, sexual, bone, metabolic and psychological health [21].

### Precocious puberty

Precocious puberty is defined as the early onset of pubertal development at an age that is >2–2.5 SD earlier than that which is expected for gender, ethnicity, and race, typically occurring at <9 years in boys and <8 years in girls [22]. The aetiology of precocious puberty can be classified as GnRH-dependent (central precocious puberty [CPP]) or GnRH-independent (peripheral precocity). CPP results from the early activation of the HPG axis, whilst peripheral precocity results from unregulated gonadal (e.g. germ-cell tumors) and adrenal production of sex-steroids or exogenous steroids [22]. Premature thelarche (PT) is characterised by isolated breast development and represents a benign variant of normal puberty. Differentiating CPP from PT is challenging due to the overlapping biochemical profile. Early and accurate diagnosis of CPP is critical for timely treatment, as early exposure to high sex-steroid concentrations results in premature epiphyseal fusion and reduced final height with associated psychosocial implications [23].

## Disorders of reproduction

In women, the pattern of GnRH secretion is essential for regulation of menstruation and reproduction. Abnormalities in the GnRH pulse frequency and amplitude are associated with several reproductive disorders, often first presenting as menstrual disturbances. Amenorrhoea is the absence of menstruation in women of reproductive age, typically defined as fewer than three menses per year, and is classified as primary (failure to initiate menarche) or secondary (abnormal cessation of menses after they have been established). Oligomenorrhoea refers to infrequent menstrual periods (intervals between menstrual cycles of more than 35 days and/or fewer than eight menses per year). Secondary amenorrhoea and oligomenorrhoea is common, estimated to affect 3–20% of women, and is associated with important health implications including subfertility [24]. The most common pathological causes of secondary amenorrhoea/oligomenorrhoea are polycystic ovary syndrome (PCOS), functional hypothalamic amenorrhoea (FHA), hyperprolactinaemia, and premature ovarian insufficiency (POI) [24].

POI is the loss of ovarian activity before the age of 40 years, which is approximately 2SD below the average age of natural menopause [25]. Causes such as chromosomal and genetic defects, autoimmune processes, chemotherapy, radiation, infection, and surgery, culminate in gonadal failure, resulting in a reduction of E2 and inhibin levels. This loss of negative feedback triggers increased gonadotrophin secretion (hypergonadotrophic hypogonadism) [25].

Whilst primary gonadal pathology underlies the pathogenesis of POI, dysregulation of pulsatile GnRH secretion is commonly observed in hyperprolactinaemia, PCOS, and FHA. In hyperprolactinaemia, raised prolactin impedes hypothalamic function through its inhibitory effect on GnRH secretion and pulsatility [26]. Consistent with this, human and rodent models with hyperprolactinaemia exhibit reduced LH pulse amplitude and frequency, which can be restored with pulsatile GnRH administration sufficient to restore fertility [27–29]. Patients with a modest rise in prolactin levels have reduced GnRH pulsatility, reflected in FSH-dominant secretion patterns. This relationship is, however, lost in the context of higher prolactin levels, which corresponded to larger macroadenomas with invasive properties, where reduced gonadotrophin secretion likely occurred secondary to structural cause as consequence of direct gonadotroph dysfunction [26].

PCOS is the most common endocrinopathy affecting women of reproductive age, with an estimated prevalence of 8–13%, depending on the diagnostic criteria used/population studied [30]. PCOS is a heterogenous condition characterised by oligo/anovulation, clinical or biochemical hyperandrogenism, and polycystic ovarian morphology on ultrasound (Rotterdam criteria requires the presence of 2 out of 3 criteria) [31]. Increased GnRH pulsatility, ovarian and adrenal steroid-dysgenesis, and metabolic dysfunction e.g. insulin resistance, are additional features frequently observed in women with PCOS [32].

In contrast, FHA is associated with reduced GnRH pulsatility; acquired functional reduction in GnRH secretion results in insufficient levels of LH and FSH to maintain folliculogenesis and ovulatory ovarian function. FHA is thus characterised by amenorrhoea with menstrual cycle length persistently >45 days or amenorrhoea of at least 3 months, history of weight loss, vigorous exercise, stress, and hypogonadotrophic hypo-oestrogenism (typically <184 pmol/L) [33]. Although both PCOS and FHA have distinct pathophysiology with recommended diagnostic criteria supported by guidelines [31,33], in practice both conditions can cause a diagnostic conundrum.

## Evaluation of the HPG axis

**Figure 1:**
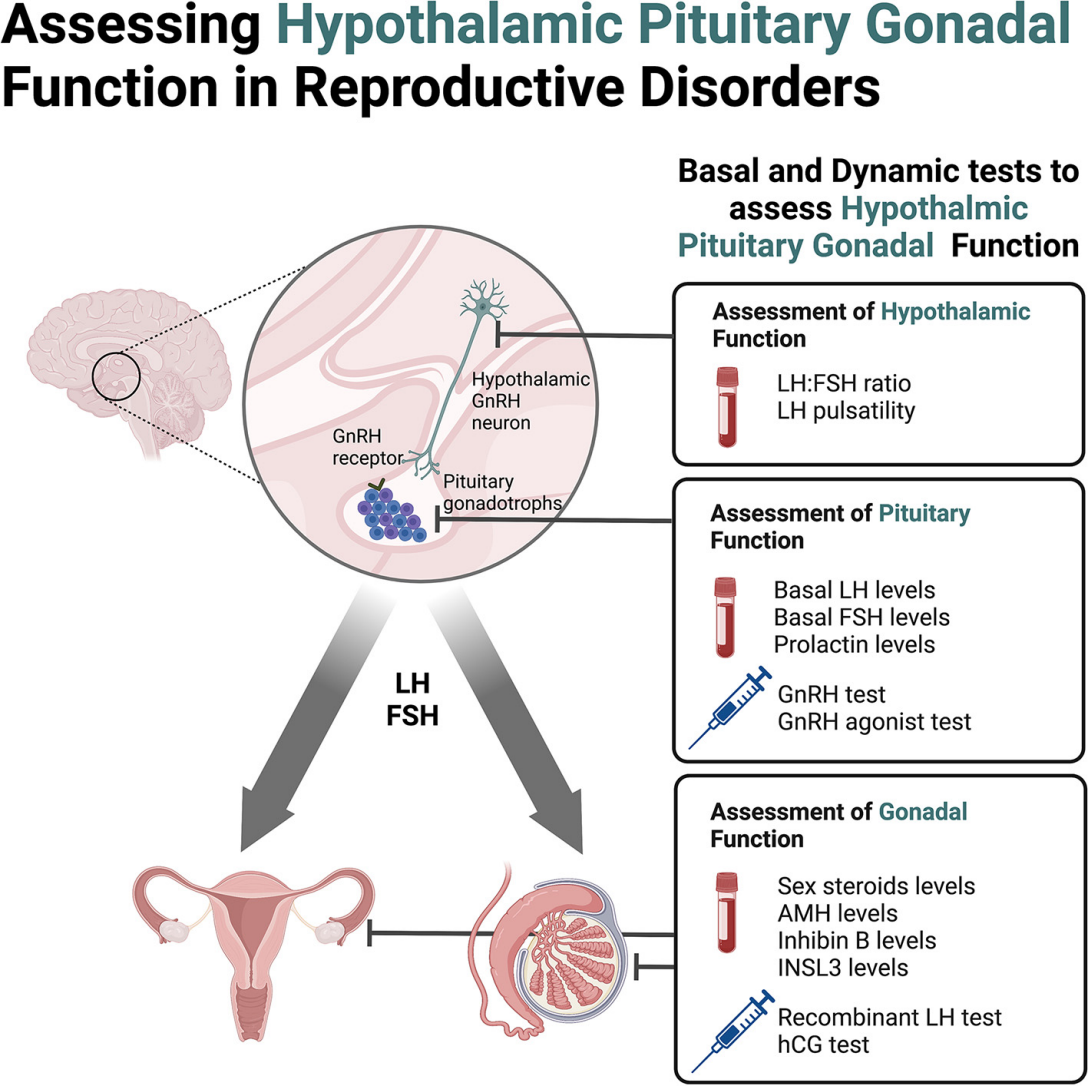
Basal and dynamic tests to assess hypothalamic, pituitary and gonadal function in reproductive disorders AMH, anti-Müllerian hormone, FSH, follicle stimulating hormone; GnRH, gonadotrophin releasing hormone; hCG, human chorionic gonadotrophin; INSL3, insulin-like peptide 3; LH, luteinising hormone.

Assessment of patients with pubertal and reproductive disorders can be aided by diagnostic tests that complement detailed medical history and clinical examination to derive accurate diagnosis and guide timely management. Quantification of key reproductive hormones, both in their basal state or as part of dynamic tests, provides insight into the function of the HPG axis (**Figure 1**). Understanding the mechanism and rationale behind these tests, some of which are already established in routine clinical practice, whilst others are performed within research capacity, can help enhance the understanding of the pathophysiology affecting hypothalamic and pituitary function in reproduction and puberty.

## Basal gonadotrophin levels

Interpretation of basal LH, FSH, in conjunction with sex-steroid levels, can aid localisation of the probable site of pathology within the HPG axis and are often first-line investigations in patients suspected to have pubertal or reproductive conditions affecting the HPG axis. For example, elevation in serum LH and FSH levels in the context of low sex-steroids (hypergonadotrophic hypogonadism) typically indicates primary hypogonadism e.g. POI as a cause of secondary oligo/amenorrhoea and primary gonadal insufficiency in delayed puberty [18]. On the contrary, low or inappropriately normal LH and FSH levels in the context of low sex-steroid levels (hypogonadotrophic hypogonadism) typically indicates hypothalamic-pituitary pathology e.g. FHA in menstrual disturbances and in delayed puberty suggests persistent (CHH), or spontaneously-resolving (CDGP) pubertal delay [18].

### Puberty

Following exclusion of primary hypogonadism in delayed puberty, basal gonadotrophin levels have been proposed to be able to distinguish CHH and CDGP. In general, patients with CHH have significantly lower basal gonadotrophin levels compared with age- and Tanner stage-matched children with CDGP, with LH appearing to be a more sensitive indicator of pubertal onset than FSH [20]. Using an ultrasensitive LH assay and a threshold of 0.65 IU/L to differentiate attainment of puberty provides a 91% sensitivity and 98% specificity to discriminate the two [34]. However, many of these studies had small study cohorts and there was substantial overlap in gonadotrophin levels between children with CDGP and CHH. Furthermore, the sensitivity of basal gonadotrophins is further reduced in individuals with partial CHH [34], which makes defining a diagnostic threshold for single basal gonadotrophin measure in the context of delayed puberty challenging [20].

Basal LH has also been evaluated as a marker for early HPG axis activation in precocious puberty. Various groups have demonstrated that judicious use of an elevated basal serum LH maybe useful in girls with suspected CPP and was highly predictive of a GnRH test result consistent with premature gonadarche, but a low LH level did not exclude central pubertal activation [35–37]. Basal LH appears to perform better than basal FSH in predicting the outcome of a GnRH stimulation test, which is considered the gold standard diagnostic test in precocious puberty [37].

### Menstrual disturbance

PCOS is associated with elevated LH pulse frequency and amplitude secondary to increased GnRH pulsatility. In 40–75% of women with PCOS, LH levels were raised to >95th percentile of healthy women [38,39]. Mean serum LH levels in women with PCOS were significantly higher (30.4 IU/L) than healthy women at all stages of the follicular phase (early follicular phase (EFP), 8.0 IU/L; midfollicular phase (MFP), 10.5 IU/L; and late follicular phase (LFP), 15.7 IU/L) [40]. Notably, LH appears to be more commonly raised in PCOS in the presence of menstrual disturbances [41]. Data from a Dutch academic centre demonstrated that when compared with FHA (*n*=159) (defined by LH <2 IU/L) and healthy controls (*n*=83), women with PCOS (*n*=3640) had elevated LH levels; 9.6 (PCOS) versus 0.8 (FHA) and 3.8 IU/L (healthy) [42]. On the other hand, women with FHA have low basal LH levels (3.2 vs 7.2 IU/L) and FSH (3.6 vs 5.0 IU/L) compared with healthy controls [43]. Basal LH levels also correlate with body weight, body mass index (BMI), and percentage of weight loss in FHA [43].

As increased GnRH pulse frequency favours LH secretion, whilst reduced GnRH frequency favours FSH secretion. Thus, the ratio between LH and FSH can provide an indirect insight into GnRH pulsatility. This has led to the premise that women with PCOS are expected to have an increased LH:FSH ratio, as PCOS is associated with increased pulse frequency. However, there are conflicting data with regards to the utility of LH:FSH ratio in establishing the diagnosis of PCOS. In one report from Vietnam, an LH:FSH ratio cut-off of 1.33 provided an area under receiver operating characteristic curve (AUROC) of 0.87 (95% CI: 0.84–0.89) to distinguish PCOS from healthy controls, and a one unit increase in LH:FSH ratio was associated with 14-fold increased odds of PCOS [44]. However, in another study, only 64% of women had an LH:FSH ratio of >2. [45,46]. No correlations were observed between the LH:FSH ratio and BMI, hirsutism (as marker of hyperandrogenism), or menstrual pattern [45,46]. As a single LH:FSH ratio only provides a single ‘snapshot’ of GnRH neuronal activity, it may fail to capture nuance in the pulsatile nature of GnRH/LH secretion. Thus, assessment of GnRH pulsatility over several hours has been advocated as a potential method of assessing hypothalamic GnRH function in patients with reproductive or pubertal disorders.

## Hypothalamic function: assessment of GnRH pulsatility

GnRH pulsatility reflects hypothalamic activity and refers to the rhythmic and intermittent release of GnRH characterised by fluctuations in pulse frequency and amplitude. Measurement of LH pulsatility is used to reflect GnRH pulsatility as peripheral sampling for GnRH cannot be conducted to reflect central hypothalamic GnRH neuronal activity in humans. Generally, LH pulsatility is predominantly only performed in research settings, as it is labour-intensive, costly to conduct and interpret.

### Puberty

Pubertal onset is heralded by increments in pulsatile GnRH secretion, which then generates the characteristic pulsatile LH secretion. Initially, this increment in gonadotrophins secretion appears to occur predominantly at night, clustered in a window immediately following sleep-onset [47]. Thus, assessment of nocturnal LH pulses has been proposed as a test in delayed puberty to signify imminent pubertal onset, with absent/reduced nocturnal LH pulses expected in children with CHH [48]. However, with increasingly sensitive LH assays and lower limits of detection, various groups have demonstrated nocturnal LH pulses in prepubertal children [20]. Wu et al. compared nocturnal LH pulses in 16 boys (mean age: 6.6 years) and 6 girls (mean age: 6.2 years) who presented prepubertally with short stature and/or delayed growth, and 8 adults with CHH (mean age: 24.1 years). In boys, there were no associations between LH pulse frequency, amplitude, and mean LH, with increasing chronological or bone age [47]. In individuals with CHH, only 25% were apulsatile, whilst 75% demonstrated 1-6 low amplitude LH pulses overnight [47]. The number of nocturnal LH pulses (male: 2.19; female: 1.83; CHH: 2.75 pulses/12 h); pulse amplitude (male: 0.16; female: 0.35; CHH: 0.19 IU/L), pulse interval (male: 168.5; female: 111.0; CHH: 124.8 min), or mean LH concentration (male: 0.10; female: 0.19; CHH: 0.14 IU/L), were not discriminatory to distinguish CHH from healthy prepubertal responses [47]. Interestingly, despite no differences in pulsatility, individuals with CHH lacked nocturnal augmentation, defined as significant increase in gonadotrophin concentrations post-sleep onset, observed in 74% of prepubertal children [47].

In precocious puberty, augmented LH secretion synchronous with sleep, consistent with that observed during puberty, is observed earlier than expected for chronological age [49]. When compared with girls with PT, pulsatile LH secretion was predominant in girls with precocious puberty whilst FSH predominates in PT [50]. However, the need for cumbersome, frequent nocturnal sampling and lack of unequivocal data on LH and FSH pulse frequency and amplitude in puberty thereby limits its applicability to routine clinical practice.

### Menstrual disturbance

As hypothalamic GnRH pulsatility is increased in PCOS and reduced in FHA, LH pulse frequency has been explored to differentiate these two most common causes of oligomenorrhoea. In the early follicular phase of healthy women, mean LH pulse frequency was 14.2 ± 4.5 pulses/24 h, and mean LH pulse amplitude was 6.02 ± 2.0 IU/L. In contrast, women with FHA demonstrated lower mean LH pulse frequency (6.75 pulses/24 h) but retained similar mean LH pulse amplitude (6.52 IU/L) [51]. Women with FHA (*n*=49) demonstrate a broad spectrum of GnRH/LH secretion patterns, ranging from a completely apulsatile pattern (8%), to abnormality of either frequency (43%) or amplitude (8%) alone, as well as combined frequency and amplitude dysfunction (27%), to normal-appearing patterns of LH secretion [51]. LH secretion patterns also fluctuated with 75% of patients meeting ≥2 different secretion patterns at different time-points in the study [51]. LH secretion patterns were not affected by E2 levels, age or duration of amenorrhoea but were affected by BMI [51]. Mean BMI in women with low frequency pattern (20.36 kg/m^2^) was significantly lower than those with low amplitude (26.34 kg/m^2^), or low amplitude with low frequency pulse patterns (23.17 kg/m^2^) [51]. However, when studied on more than one occasion, women who initially had low frequency pulse pattern on their first study, over time demonstrated various LH secretion patterns in the absence of any significant weight change [51]. Whilst most healthy women have reduced LH pulsatility during sleep, in 45% of women with FHA, nocturnal augmentation was observed [51]. Pulse amplitude was increased in 71% of women who demonstrated nocturnal augmentation from 5.0 IU/L to 12.2 IU/L. In another longitudinal study conducted over 9 months in 14 women with FHA, LH pulse frequency, amplitude and mean LH were increased at night [52]. However, daytime LH pulse frequency, amplitude and mean LH appeared to be more consistent during repeated studies (93% of LH pulse frequency varied by <2 pulses per 8 h) whereas nocturnal LH secretion pattern demonstrated greater variability [52].

Women with PCOS have an inherent abnormality in the GnRH pulse generator independent of sex-steroids, with increased LH pulse frequency [53] and amplitude [40] compared with healthy women at all three stages of the follicular phase [40]. Mean LH pulse frequency in women with PCOS was significantly higher than controls (24.8 vs 15.6–22.2 pulses/24 h). Similarly, mean LH pulse amplitude in women with PCOS (13.6 IU/L) was nearly twice that in healthy women at all stages of the follicular phase (EFP, 6.5 IU/L; MFP, 5.1 IU/L; LFP, 7.2 IU/L) [40]. LH pulse amplitude is higher in lean PCOS (BMI<23 kg/m^2^, 13.3 IU/L) than obese PCOS (BMI>30 kg/m^2^, 6.4 IU/L), or healthy controls (5.3 IU/L) [54]. The mean LH pulse amplitude in PCOS correlated with mean LH level (*r* = 0.89) and LH:FSH ratio (*r* = 0.72), but not with gonadal steroid levels. Taken together, LH pulse frequency is increased in most women with PCOS by ∼40% and reduced in 78% of women with FHA [55]. As LH pulse amplitude is not increased in obese PCOS [54] and reduced in only 43% of FHA, LH pulse frequency is likely to have greater discriminatory potential in differentiating PCOS and FHA than LH pulse amplitude [55].

In women with hyperprolactinaemia (*n*=7), mean LH pulse frequency was significantly lower than in healthy controls during the early follicular phase (7.6 vs 15.4 pulses/24 h) [56]. Mean LH amplitude in hyperprolactinaemic women prior to treatment (5.2 IU/L) was significantly higher compared with healthy women during the follicular phase (3.5 IU/L). Following bromocriptine therapy and resumption of menses, the LH pulse frequency incremented to 10.2 pulses/24 h and the LH pulse amplitude reduced to average of 3.9 IU/L. However, the patterns of LH pulses demonstrated marked intra- and inter-individual variability prior to bromocriptine treatment, e.g., LH pulse amplitude ranged from 1.0 to 23.0 IU/L with prolonged periods of low LH secretion interspersed with periods of normal or increased LH secretion. During and following bromocriptine therapy, both LH frequency and amplitude became more uniform (post-treatment LH amplitude: range 1.6–10.2 IU/L), and were more representative of healthy women at the same stage of the menstrual cycle (LH amplitude: range 1.2–10.4 IU/L) [56].

## Pituitary function: GnRH test and GnRH agonist (GnRHa) test

Given the diagnostic limitations of basal gonadotrophins, providing a ‘snap-shot’ of GnRH neuronal activity and the cumbersome nature of pulsatility studies, many clinicians employ dynamic stimulation tests to aid the distinction of pubertal and reproductive conditions. GnRH and GnRH agonist (GnRHa) tests directly stimulate pituitary gonadotrophs; differential LH and FSH response to these stimulation tests can help discriminate different reproductive and pubertal disorders.

### Puberty

Intravenous GnRH induces a dose-dependent increase in gonadotrophins both in prepubertal and pubertal adolescents [20]. The use of GnRH stimulation in delayed puberty relies on the premise that the gonadotrophin response to GnRH or GnRHa will be greater in CDGP than in CHH, as individuals with CDGP have previously been exposed to endogenous GnRH whilst individuals with CHH have not been exposed to endogenous GnRH or lack functional GnRH receptors [20]. In general, adolescents with CHH have lower stimulated LH levels compared with those with CDGP but unfortunately, significant variability in gonadotrophin response means that up to 30% have peak LH responses indistinguishable from those with CDGP [34,57].

The half-life of GnRH is 2–4 mins [58] attributed to the degradation of the glycine-leucine bone between amino acids 6 and 7. To circumvent this, several GnRH synthetic analogues, characterised by alterations in the amino acid in position 6, have been developed resulting in increased potency and half-life due to greater affinity to GnRH receptors and resistance to enzyme degradation [59]. The greater stimulatory effects of GnRHa have been postulated to enable better discrimination between CDGP and CHH, as a greater stimulus would result in activation of primed gonadotrophs in patients with CDGP [20]. Various GnRHa compounds have been used to differentiate CDGP and CHH including nafarelin [60], triptorelin [61], buserelin [62], and leuprolide [63]. Although the performance of GnRHa stimulation appeared to be superior and more robust compared with GnRH, the lack of consistency and overlap in diagnostic thresholds limits its reliability in delayed puberty [20].

Though the GnRH test remains the gold standard diagnostic test for CPP [64], GnRHas such as triptorelin and leuprorelin acetate have been used as alternative stimulation agents. A randomised controlled trial compared the diagnostic accuracy of subcutaneous 0.1mg/m^2^ triptorelin versus intravenous GnRH 100 μg/m^2^, in girls with suspected CPP (*n*=46). Using a maximal LH response threshold at 3hrs post-triptorelin of >7 IU/L by immunofluorometric assay (IFMA), or >8 IU/L by electrochemiluminescence immunoassay (ECLIA), confirmed the diagnosis of CPP with a specificity of 100% (95% CI: 75–100%) and sensitivity 76% (95% CI: 58–89%) [65]. LH at 3 h post-triptorelin showed a significant correlation with the existing gold standard (peak LH post-GnRH stimulation) [65]. The use of GnRHa stimulation in the diagnosis of CPP is a subject of ongoing research as, at present, there is paucity of quality data, consensus on timings, and LH cut-off values (appear to vary between studies depending on the GnRHa assessed) [66].

### Menstrual disturbance

There were no significant differences in the LH rise after 50 µg GnRH injection between women with FHA (1.8 → 11.0 IU/L; *n*=8) and healthy women in the early follicular phase (2.7 → 12.9 IU/L; *n*=6), or luteal phase (2.3 → 16.6 IU/L; *n*=9) [67]. Interestingly, women with FHA demonstrated a more pronounced FSH-response to 50 µg GnRH injection compared with healthy women in the luteal phase (3.1 → 8.6 IU/L vs 2.5 → 5.2 IU/L) [67]. Despite women with anorexia nervosa (average BMI: 15.1 kg/m^2^; *n*=40) having lower basal LH (3.2 vs 7.2 IU/L) and FSH (3.6 vs 5.0 IU/L) levels than healthy controls, LH rises following 100 μg GnRH were similar [43]. However again, FSH responses were more marked in women with anorexia nervosa compared with healthy controls (AUC for FSH in anorexia 11.13 IU/L/min vs healthy controls 6.98 IU/L/min) [43]. LH responses to GnRH stimulation positively correlated with BMI (*r* = 0.341), bodyweight (*r* = 0.382), and inversely with % of weight loss (*r* = −0.374) [43].

Absolute rises in LH after GnRH (dose range: 2–20 μg) were two- to three-fold greater in PCOS (BMI: 34.7 kg/m^2^; *n*=13) than in healthy women (BMI: 26.8 kg/m^2^, *n*=13) [68]. Notably, LH responses to GnRH positively correlated with basal LH values; therefore, due to higher basal LH values in PCOS (7.5 ± 1.2 vs 3.6 ± 0.4 IU/L), the percentage rise in LH after GnRH was not increased [68]. Maximal LH responses to GnRH positively correlated with basal E2 levels and inversely to BMI [68]. In women with PCOS, LH concentrations at baseline and following GnRH stimulation were higher than controls – 0 min: PCOS, 9.09 vs control, 4.83 IU/L; 30 min: PCOS, 35.48 vs control, 16.30 IU/L; 60 min: PCOS, 33.86 vs controls 13.45 IU/L [69]. No significant difference in baseline or stimulated FSH concentrations were observed between the two groups [69].

Gonadotrophin responses to subcutaneous triptorelin (GnRHa) 0.2 mg have recently been described in a single-blinded placebo-controlled study of 9 eumenorrhoeic women, 6 women with PCOS, and 6 women with FHA [70]. LH response in women with FHA appeared to be expedited compared with healthy women or women with PCOS (mean LH change at 1 h: 8.8 IU/L in controls, 29.1 IU/L in FHA and 12.7 IU/L in PCOS) [70]. LH levels reached a maximum peak concentration at ∼4 h after triptorelin administration; peak LH stimulation was greatest in healthy women and lowest in women with FHA (mean LH change at 4 h 45.7 IU/L in healthy women, 31.3 IU/L in FHA, and 36.9 IU/L in PCOS), however the AUC of change in serum LH following triptorelin administration over 10 h did not differ between groups [70]. Interestingly, the change in serum FSH was markedly reduced in women with PCOS compared with healthy women and women with FHA [69]. Similarly, in women undergoing oocyte donation with at least one polycystic morphology ovary (*n*=60) had a lower maximal change in serum FSH at 4hrs post-triptorelin (dose range: 0.2–0.4 mg) than in women with normal morphology ovaries (*n*=91) (mean FSH levels at 4 h 34.1 vs 42.3 IU/L) [70].

Dysregulation of steroidogenesis and androgen secretion causing hyperandrogenism is hypothesised to contribute to the pathophysiology and clinical hallmarks of PCOS. In 1989, Barnes et al. first reported generalised ovarian steroidogenic hyperresponsiveness to GnRHa (nafarelin) stimulation independent of whether pre-treatment with dexamethasone was used to eliminate confounding adrenal steroidogenesis [71]. In women with PCOS (*n*=8), LH responses, and ovarian steroid intermediates – 17-hydroxyprogesterone (17-OHP) and androstenedione levels were significantly higher than in controls (*n*=16) in response to nafarelin [71], postulated to be secondary to abnormal regulation of 17-hydroxylase and 17,20-lyase activities within the theca cells. As the ovaries and adrenal cortices contribute approximately equivalent amounts of androgen and androgen precursors in response to their trophic hormones, LH and ACTH respectively; localisation of the source of androgen excess (ovaries/adrenals/both) is important to our understanding of the pathophysiology of androgenic excess in PCOS [32].

To ascertain that the hyperandrogenaemia in PCOS was of ovarian origin (termed ‘functional ovarian hyperandrogenism’ [FOH]), stimulation tests that provide direct ovarian stimulation including the GnRHa, and human chorionic gonadotrophin (hCG) [72] and recombinant LH (rLH) tests [73] have been used to quantify the response of ovarian steroid intermediates as surrogate markers of ovarian steroidogenic activity, or indirectly using ‘dexamethasone androgen-suppression test’ (DAST) to suppress adrenal steroidogenesis [74], which are further explored in the Gonadal Function – basal markers and dynamic tests section.

## Gonadal function: basal markers and dynamic tests

Various basal and dynamic tests of gonadal markers have been investigated to aid the differentiation of reproductive and pubertal disorders. Clinical applications and the utility of these tests in the context of disorders of puberty and reproduction are explored below.

### Inhibin B (INB)

In delayed puberty, inhibin B (INB) has been evaluated as a potential marker to differentiate CDGP and CHH. INB is a glycoprotein heterodimer which is secreted by and is reflective of the number and function of Sertoli cells in prepubertal boys. In adulthood, INB closely reflects testicular mass, which comprises germ cells and Sertoli cells, and reflects the spermatogenic capability. INB is therefore typically reduced in men with CHH. Indeed, in adult men with CHH, testicular volume correlated positively with INB [75], this stands to reason, as INB is reflective of Sertoli cell function, which comprises the majority of testicular volume [21]. In one study, INB was higher in boys with CDGP than boys with CHH (87.6 vs 19.8 pg/ml), giving a AUROC for the diagnosis of CDGP of 0.955, at a recommended threshold of ≥28.5 pg/ml [76]. Other groups have described the use of INB in combination with basal LH to improve diagnostic accuracy; the combination of LH <0.3 IU/L with INB <111 pg/ml increased the specificity to 98.1%, whilst basal LH and INB when used in isolation provided specificities of 88% and 92%, respectively [61].

In females, INB is a product of granulosa cells, which stimulates theca cell androgen production and suppresses FSH secretion. INB levels fluctuate during the menstrual cycle and peak during the mid-follicular phase [77]. Mean serum INB was lower in women with FHA compared with controls (48 vs 87 pg/ml) [78]. In PCOS, INB levels demonstrated more heterogeneity than in FHA, with some studies reporting higher INB while others found no difference between PCOS and controls [55]. For example, a cross-sectional study revealed no difference in INB between PCOS (*n*=50) and healthy women (*n*=25) in the early follicular phase (94.2 vs 75.2 pg/ml) despite the presence of larger ovarian volume and a greater number of follicles in women with PCOS [79]. INB correlated negatively with BMI (*r* = −0.413), fasting insulin (*r* = --0.409) but positively with LH pulse amplitude (*r* = 0.512) and LH pool (*r* = 0.419) [79]. In women with PCOS, administration of hCG results in reduction of INB levels (pre-hCG 223.8 vs post-hCG 152.4 pg/ml), whilst diazoxide (which blocks insulin secretion) results in significant increase in INB levels (pre-diazoxide 85.4 vs post-diazoxide 136.6 pg/ml) [79]. Direct manipulation of LH through hCG stimulation demonstrates that, although a positive correlation between LH and INB exists, there is no cause-and-effect relationship between acute hCG (LH) stimulation and INB secretion. On the contrary, suppression of insulin secretion resulted in increased INB levels, consistent with the hypothesis that insulin negatively regulates INB and may explain the inverse relationship between BMI and INB in women with obesity and raised insulin levels [79].

### Insulin-like peptide 3 (INSL3)

In males, insulin-like peptide 3 (INSL3) reflects the number, and degree of differentiation of Leydig cells [80]. The median INSL3 was 1.08 ng/ml (0.95, 1.38) in eugonadal men and 0.05 ng/ml (0.01, 0.18) in men with CHH, whilst the median INSL3 was 0.35 ng/ml (0.24, 0.47) in boys with CDGP and 0.15 ng/ml (0.14, 0.21) in boys with CHH [75]. INSL3 was found to provide better discriminatory potential between eugonadal adult men and men with CHH (AUROC: 100%) than between boys with CDGP and those with CHH (AUROC: 86.7%) [75]. These data are consistent with INSL3 being more reflective of the attainment of complete pubertal development, whereas INB appears to have greater predictive power in the setting of boys with delayed puberty. INSL3 is not acutely regulated in the short-term (hours) by gonadotrophins but is dependent on Leydig cell proliferation and differentiation; it reflects a chronic differentiation dependent process (several days) [81] and therefore changes slower than INB [80].

In females, INSL3 is produced by the theca interna cells of growing antral follicles and corpora lutea [82]. INSL3 acts via its G-protein-coupled receptor, RXFP2, leading to androstenedione production and its conversion by granulosa cells into oestrogens, which in turn create a positive feedback loop promoting the expression of more theca cell INSL3 [82]. Whilst INSL3 levels in healthy men range between 0.5 and 2.0 ng/ml, in women of reproductive age, INSL3 levels usually range between 0.01 and 0.1 ng/ml (maximum ∼0.2 ng/ml) [82]. INSL3 levels fluctuate during the menstrual cycle, wherein it is minimal around menstruation and increases during the early to mid-follicular phase by 2- to 3-fold, representing follicle growth [83]. Therefore, INSL3 should be assessed at a specific stage of the menstrual cycle. A cross-sectional study of 909 women presenting with subfertility found similar INSL3 levels amongst women with fallopian pathology and subfertility related to male factor who were assumed to have healthy ovarian function (0.079 ng/ml, *n*=277 vs 0.073 ng/ml, *n*=67) [83]. In contrast, women with PCOS (*n*=134) had significantly elevated INSL3 (0.104 ng/ml) whilst those with low ovarian reserve (*n*=219; defined as age-matched AMH <10th percentile) had significantly reduced INSL3 (0.058 ng/ml) [83]. However, in women with PCOS with overweight/obesity, INSL3 was not significantly higher than in controls [84]. INSL3 positively correlated with total and free testosterone, LH levels, 17-OHP response to buserelin, hirsutism, androgen levels, and ovarian follicle number [84]. At present there is a paucity of data on INSL3 levels in FHA, however as INSL3 is produced by the theca cell, one would anticipate lower INSL3 secondary to reduced gonadotrophin stimulus to theca cell [55].

### Anti-Müllerian hormone (AMH)

Anti-Müllerian hormone (AMH) is produced by the granulosa cells of growing antral follicles; AMH levels therefore can be regarded as a surrogate marker of antral follicle count (AFC) [85]. Raised serum AMH >60 pmol/L increased the odds of oligo/amenorrhoea 28.5-fold compared with those with AMH <15 pmol/L and predicted menstrual disturbance with an AUROC of 0.77 [85]. Although AMH is markedly increased in PCOS, and corresponded to the number of PCOS features (median AMH in women with all three features 65.6 vs 14.6 pmol/L in women with no PCOS features [85]), women with FHA can also have mildly elevated AMH levels. For example, median AMH was higher in age-matched women with PCOS (47.9 pmol/L), than in FHA (27.1 pmol/L), or in healthy women (13.6 pmol/L) [42]. Thus, whilst AMH has discriminatory potential to distinguish PCOS from healthy women, its performance could be tempered in women with menstrual disturbance [55].

Menopause is a stage in a woman’s reproductive life characterised by depletion of ovarian reserve and cessation of menstruation. The age at which menopause occurs is an important determinant of adverse health outcomes, for instance women with POI (menopause onset <40 years) or women who experience early menopause (menopause onset between 40 and 45 years) are at increased risk of overall mortality, cardiovascular disease, osteoporosis, predisposition to Type 2 diabetes as well as infertility [86]. Currently menopause is a retrospective diagnosis made following 12 consecutive months of amenorrhoea or 4 months with increased FSH concentrations [25]. Health implications associated with menopause, particularly early menopause and POI, have garnered interests around markers that can facilitate early diagnosis or prediction of menopause to enable earlier interventions. Undetectable AMH was strongly associated with menopause stage, had equivalent accuracy to elevated FSH in established menopause and declines in advance of elevated FSH concentrations. A recent systematic review demonstrated strong associations between the risk of early menopause and low AMH levels particularly when adjusted for age in younger women [87]. For example, an AMH of 0.1 ng/dL at age 20 years predicts an age of menopause of 33 years (95% CI: 27-36), but at 34 years predicts an age of menopause of 41 years (95% CI: 34–46) [88]. In the context of POI, AMH levels are significantly lower/undetectable compared with age-matched controls, and correlate positively with follicle number. In a study of 1,998 women aged under 40 years with different ovarian reserve, subdivided into control with normal ovarian reserve (NOR, *n*=987), pre-POI (*n*=410; FSH>10 IU/L but ≤25 IU/L), early POI (*n*=147; FSH >25 IU/L but ≤40 IU/L), and ‘premature ovarian failure’ (*n*=454; defined as FSH >40 IU/L) group, an AMH of ≤0.25 ng/ml was diagnostic of POI with a sensitivity of 92.46% and specificity of 90% [89]. AMH also showed good discriminatory value from other causes of menstrual disturbances e.g. PCOS, FHA and hyperprolactinaemia, where AMH levels are characteristically normal or high in these conditions. Predictive value of AMH increases with age, however the wide confidence intervals with both single and serial AMH measurements in younger women currently limits its applicability in clinical practice to predict time to menopause [87].

## Dynamic tests of gonadal function

### Human chorionic gonadotrophin (hCG) in puberty

Dynamic tests utilised to assess gonadal function such as with hCG stimulation have been used to assess for the presence of functioning testicular tissue and testosterone biosynthesis. hCG stimulation test utilises the ability of hCG as LH receptor agonist to increase androgen production in Leydig cells. In delayed puberty, testosterone response to hCG stimulation is postulated to be higher in CDGP (with previous exposure to gonadotrophins) compared with CHH (absent preceding exposure to gonadotrophins). Various protocols with different hCG doses, durations of test, numbers of injections, have been described with predicted values for diagnostic accuracy between 82 and 86% [20].

### Human chorionic gonadotrophin (hCG) in PCOS

Unlike the GnRHa test, which evaluates the combined effect of LH and FSH, both hCG and rLH directly stimulate the LH receptor on theca cells. HCG stimulation led to significant increase in serum 17-OHP, androstenedione, free testosterone, and E2, with no effect seen of FSH pre-treatment (FSH pre-treatment decreased free testosterone in eumenorrhoeic controls) [72]. The response to hCG was preserved even after prolonged pituitary suppression with GnRHa [90]. FOH, therefore, is likely to be secondary to a combination of intrinsic thecal hypersensitivity to LH and paracrine defect in FSH inhibition of theca cell function and hence androgen production in women with PCOS [90].

### Recombinant LH (rLH) in PCOS

Although hCG and rLH both bind the LH chorionic gonadotrophin receptor (LHCGR), they induce distinct intracellular signalling and steroidogenic profile. HCG induces more potent signaling with respect to cAMP and reached the maximal effect significantly faster upon binding to LHCGR than rLH [91]. Different intracellular signalling through activation of cAMP/PKA by hCG and preferential phosphorylation of ERK1/2 and AKT by rLH accounts for the difference in steroidogenic activity elicited [92]. The GnRHa and hCG stimulation tests previously described involve supraphysiological and prolonged ovarian stimulation; for instance, plasma LH approaches or exceeds 100 IU/L within 4hrs following acute GnRHa administration [71], and hCG protocols utilise 5000–10,000 IU hCG, which results in potent and prolonged ovarian stimulation [72]. Intermittent (pulse-like) rLH stimulation to provide a near-physiological LH stimuli was utilised to evaluate effect of androgen secretion in PCOS (*n*=7) compared with controls (*n*=13) [73]. Intravenous infusion of rLH following pre-treatment with GnRH antagonist (to block endogenous LH secretion) and dexamethasone (to block adrenal androgen secretion) in women with PCOS results in significant increase in the mean plasma 17-OHP (0.78 vs 0.33 ng/ml) and testosterone (0.86 vs 0.32 ng/ml) compared with controls [73]. Furthermore, linear regression of 17-OHP on LH yielded a higher mean slope of 0.028 in PCOS vs 0.005 in controls; this difference remained significant even after excluding data from the supraphysiological rLH dose (300 IU) [73]. These results are consistent with previous data from GnRHa and hCG stimulation studies, supporting the notion of exaggerated 17-OHP response to LH stimulation in women with PCOS (Table 1 summarises the hypothalamic, pituitary, and gonadal markers frequently observed in disorders of puberty and reproduction compared with controls.)

**Table 1:** Hypothalamic, pituitar,y and gonadal markers in disorders of puberty and reproduction

	Hypothalamic markers	Pituitary markers	Gonadal markers
*Disorders of Puberty*			
Constitutional delay of growth and puberty (CDGP)	Basal LH ↓	GnRH test ↓	Inhibin B ↓
	LH pulsatility** ↔	GnRHa test↓	INSL3** ↓
			hCG test ↓
Congenital hypogonadotrophic hypogonadism (CHH)	Basal LH ↓↓	GnRH test ↓↓	Inhibin B ↓↓
	LH pulsatility** ↓/↔*	GnRHa test ↓↓	INSL3** ↓↓
			hCG test ↓↓
*Disorders of Reproduction*			
Functional hypothalamic amenorrhoea (FHA)	Basal LH ↓	GnRH test ↔/↑^†^	Inhibin B ↓
	LH:FSH ratio ↓/↔	GnRHa test ‡	AMH ↑
	LH pulsatility** ↓/(↔)		
Polycystic ovary syndrome (PCOS)	Basal LH ↑	GnRH test ↑^¤^	Inhibin B ↓/↑^§^
	LH:FSH ratio ↑	GnRHa test ‡	INSL3** ↔/↑
	LH pulse frequency** ↑		AMH ↑↑
Hyperprolactinaemia	Basal LH ↓/↔	Prolactin ↑↑	
	LH pulsatility** ↓		

Arrows indicate the responses frequently observed in disorders of puberty and reproduction compared with controls. However as explored in the manuscript, the lack of diagnostic threshold, considerable overlaps between conditions e.g. FHA vs PCOS, are limitations of these diagnostic markers.

*Lacks nocturnal augmentation; ^†^Pronounced FSH response following GnRH stimulation compared with controls; ^¤^ Due to higher basal LH values in PCOS the relative rise in LH after GnRH was not increased; ^§^Heterogenous results affected by other factors e.g. BMI.

‡ LH response expedited compared with healthy women and women with PCOS, peak LH stimulation was greatest in healthy women and lowest in women with FHA but AUC of change in serum LH following triptorelin administration over 10 hours did not differ between groups.

**Hypothalamic, pituitary and gonadal markers frequently measured in research capacity AMH, anti-Müllerian hormone, FSH, follicle stimulating hormone; GnRH, gonadotrophin releasing hormone; hCG, human chorionic gonadotrophin; INSL3, insulin-like peptide 3; LH, luteinising hormone.

Suppression of the ACTH-dependent adrenocortical androgen pathway by DAST indirectly assesses ovarian androgenic function as inappropriately elevated serum testosterone post-DAST suggests an ACTH-independent source of androgen likely to be of ovarian origin [74]. The vast majority (87%) of women with PCOS have FOH; two-thirds are characterised by 17-OHP hyperresponsiveness to GnRHa or hCG stimulation (functionally typical FOH), whilst two-thirds of the remainder have FOH detectable by DAST (functionally atypical FOH) [32]. Severe hyperandrogenism, impaired glucose tolerance, and diabetes mellitus, appeared to be more prevalent in ‘functionally typical FOH’ than those with ‘functionally atypical FOH’ [32]. However, it remains to be demonstrated whether deciphering these pathophysiologic categorisations has clinical value [32].

## Future perspectives on basal and dynamic tests

### Circulating Kisspeptin levels

As kisspeptin neurons regulate GnRH neuronal activity, circulating levels of kisspeptin have been proposed to reflect GnRH neuronal pulsatility (although the origin of circulating levels in these studies has yet to be confirmed to be central). To date, kisspeptin levels are conducted solely in research settings and many commercial kisspeptin assays are not consistently reliable especially at low concentrations (i.e., in non-pregnant women).

### FHA

Amongst women with FHA, kisspeptin levels were lower in women with LH ≤3 IU/L compared with those with LH >3 IU/L at 1.7 ng/ml vs 2.6 ng/ml, respectively [93]. Temporal relationship between kisspeptin and LH pulsatile secretion was also observed, suggesting that both hormones are co-secreted and consistent with a central source [93]. Kisspeptin inversely correlated with physical activity (*r* = -0.41), but positively with BMI and fat mass [94].

### PCOS

Most studies have reported higher kisspeptin levels in women with PCOS [95] and a meta-analysis determined that raised circulating kisspeptin levels predicted PCOS diagnosis (AUROC of 0.84) [96]. Women with PCOS and oligomenorrhoea (defined as menstrual interval >45 days) had significantly higher kisspeptin pulse frequency and concentrations compared with the eumenorrhoeic PCOS group [97], however the effect of BMI on kisspeptin levels was not significant [98].

### Puberty

Circulating kisspeptin levels were first evaluated as a potential marker for precocious puberty in 2009 [99]. Serum kisspeptin levels in girls with CPP were found to be significantly higher than in age-matched prepubertal controls (14.62 ± 10.2 pmol/L vs 8.35 ± 2.98 pmol/L) [99], however there was some overlap between the two groups [99]. A recent meta-analysis of 316 CPP patients and 251 controls demonstrated higher kisspeptin levels in the CPP cohort compared with healthy controls, with a bias-corrected standardised mean difference (SMD) 1.53 (95% CI: 0.56–2.51) [100]. Subgroup analyses demonstrated a positive correlation between serum kisspeptin and age in the CPP cohort and association between serum kisspeptin levels and precocious thelarche [100].

### Dynamic response to kisspeptin challenge in delayed puberty/CHH

Gonadotrophin responses to kisspeptin-10 [101,102] and kisspeptin-54 [103] have recently been described in paediatric and adult cohorts with CHH. The ability of kisspeptin to directly stimulate GnRH release offers a novel probe into hypothalamic GnRH function. As hypothalamic GnRH neuronal migration/secretion/function are impaired in CHH, it is postulated that administration of exogenous kisspeptin to patients with CHH would yield no or attenuated GnRH-induced gonadotrophin response.

In adult men with CHH (*n=*21), kisspeptin-54 led to a maximal LH rise of 0.4 IU/L compared with 12.5 IU/L in eugonadal men (*n*=21) [103]. LH response to kisspeptin-54 had an AUROC of 1.0 (95% CI: 1.0–1.0), performing better in differentiating men with CHH from eugonadal men than a GnRH stimulation test (AUROC: 0.88, 95% CI: 0.76–0.99) [103]. LH responses to kisspeptin-54 within the CHH cohort with anosmia or those with pathogenic/likely pathogenic variants in CHH genes were also significantly lower compared with other men with CHH suggesting functional correlation [103].

In a paediatric cohort, LH responses to kisspeptin-10 stimulation were shown to be able to accurately predict progression through puberty in 16 children (3 girls and 13 boys) with delayed or stalled puberty after longitudinal follow-up [101,102]. All children (*n=*8) in the kisspeptin-responder group (defined as LH response of ≥0.8 IU/L) subsequently progressed through puberty, in contrast kisspeptin non-responders (*n*=8) with LH response to kisspeptin of ≤0.4 IU/L who did not develop physical signs of puberty by 18 years. The kisspeptin-stimulation test was found to predict pubertal outcomes more accurately than other tests utilised in delayed puberty such as inhibin B, overnight LH, GnRH stimulation and genetic testing [102].

MVT-602 (formerly known as TAK-448) is a nanopeptide KISS1R agonist that has enhanced stability, potency and water solubility [104]. MVT-602 induces an LH rise of a similar amplitude to the LH rise following kisspeptin-54 consistent with their analogous mechanism of action through stimulation of the KISS1R on hypothalamic neurons. However, the timing of peak LH attained was later after MVT-602 than after kisspeptin-54 [105]. When subcutaneous bolus of 0.03 nmol/kg of MVT-602 was administered to healthy women and women with PCOS and FHA, exaggerated and early LH rise was seen in women with FHA; this was not observed in healthy women or women with PCOS [105]. This was postulated to be due to up-regulation of KISS1R in women with FHA augmenting the LH response. Further studies are needed to determine if the use of a kisspeptin challenge test can be used to delineate the endocrine profile of other reproductive conditions centred around hypothalamic dysfunction.

## Conclusion

Disorders of puberty and reproduction are common, often with associated important health implications and considerable psychosocial impact for those affected and their families. Securing an accurate diagnosis in a timely manner is, therefore, invaluable to instigate optimal management to address current symptoms, minimise long-term health implications and ensure patients and their families are counselled appropriately.

Markers of hypothalamic, pituitary and gonadal function, both in their basal state and as part of dynamic tests, complement detailed medical history, clinical examination and contribute to confirm or refute diagnoses. Unfortunately, for many conditions, no gold standard investigation has been established to unequivocally distinguish important conditions of delayed puberty such as CDGP and CHH and common reproductive disorders including FHA and PCOS. Lack of consensus on diagnostic thresholds, compounded by assay variabilities, and use of different compounds in stimulation tests, make differentiating these conditions a challenge in practice. To circumvent these limitations, various groups have advocated combination of different markers to improve diagnostic yield and acceptability of these tests for patients, e.g., INB and basal LH to avoid need for GnRH/GnRHa stimulation test in delayed puberty. Feasibility of proposed diagnostic tests is an important consideration prior to translation to clinical practice; LH pulsatility test for example is both cumbersome and labour intensive, hence has limited utility in real-world clinical practice and is therefore predominantly conducted in the research setting.

This review explored conditions affecting the hypothalamic and pituitary function in reproduction and puberty together with the challenges faced with the currently available baseline and dynamic tests used to interrogate hypothalamic, pituitary, and gonadal function. We hope to highlight the need for future studies and diagnostic markers to better classify, and in turn further our understanding of the hypothalamic pituitary gonadal function in reproductive and pubertal disorders.

## Data Availability

Data sharing is not applicable to this review

## Open Access

Open access for this article was enabled by the participation of Imperial College London in an all-inclusive *Read & Publish* agreement with Portland Press and the Biochemical Society under a transformative agreement with JISC.

## Abbreviations

ACTH: adrenocorticotrophic hormone
AMH: anti-Müllerian hormone
ARC: arcuate nucleus
AUROC: area under receiver operating characteristic curve
AVPV: anteroventral periventricular nucleus
BMI: body mass index
CDGP: constitutional delay of growth and puberty
CHH: congenital hypogonadotrophic hypogonadism
CPP: central precocious puberty
DAST: dexamethasone androgen-suppression test
E2: oestradiol
EFP: early follicular phase
ERα: oestrogen receptor α
FHA: functional hypothalamic amenorrhoea
FOH: functional ovarian hyperandrogenism
FSH: follicle-stimulating hormone
GnRH: gonadotrophin-releasing hormone
GnRHa: gonadotrophin-releasing hormone agonist
GnRHR: gonadotrophin-releasing hormone receptor
hCG: human chorionic gonadotrophin
HPG: hypothalamic–pituitary–gonadal
INB: inhibin B
INSL3: insulin-like peptide 3
LFP: late follicular phase
LH: luteinising hormone
LHCGR: LH chorionic gonadotrophin receptor
MFP: midfollicular phase
MKRN3: makorin RING finger protein 3
PCOS: polycystic ovary syndrome
POI: premature ovarian insufficiency
PT: premature thelarche
rLH: recombinant luteinising hormone
RP3V: rostral periventricular area of the third ventricle
17-OHP: 17-hydroxyprogesterone
